# Supplementary material for: (p)ppGpp-dependent activation of gene expression during nutrient limitation
Source: mBio. 2025 Aug 18;16(9):e01288-25. doi: 10.1128/mbio.01288-25 (PMC12421865; doi:10.1128/mbio.01288-25)
Supplement: Supplemental material — Supplemental figures and tables. [file mbio.01288-25-s0001.pdf]

SUPPLEMENTAL MATERIAL

(p)ppGpp-dependent activation of gene expression during nutrient limitation

Supplemental Figures S1 to S11

Supplemental Figures Legends S1 to S11

Supplemental Table S1 to S3

Supplemental Figure S1

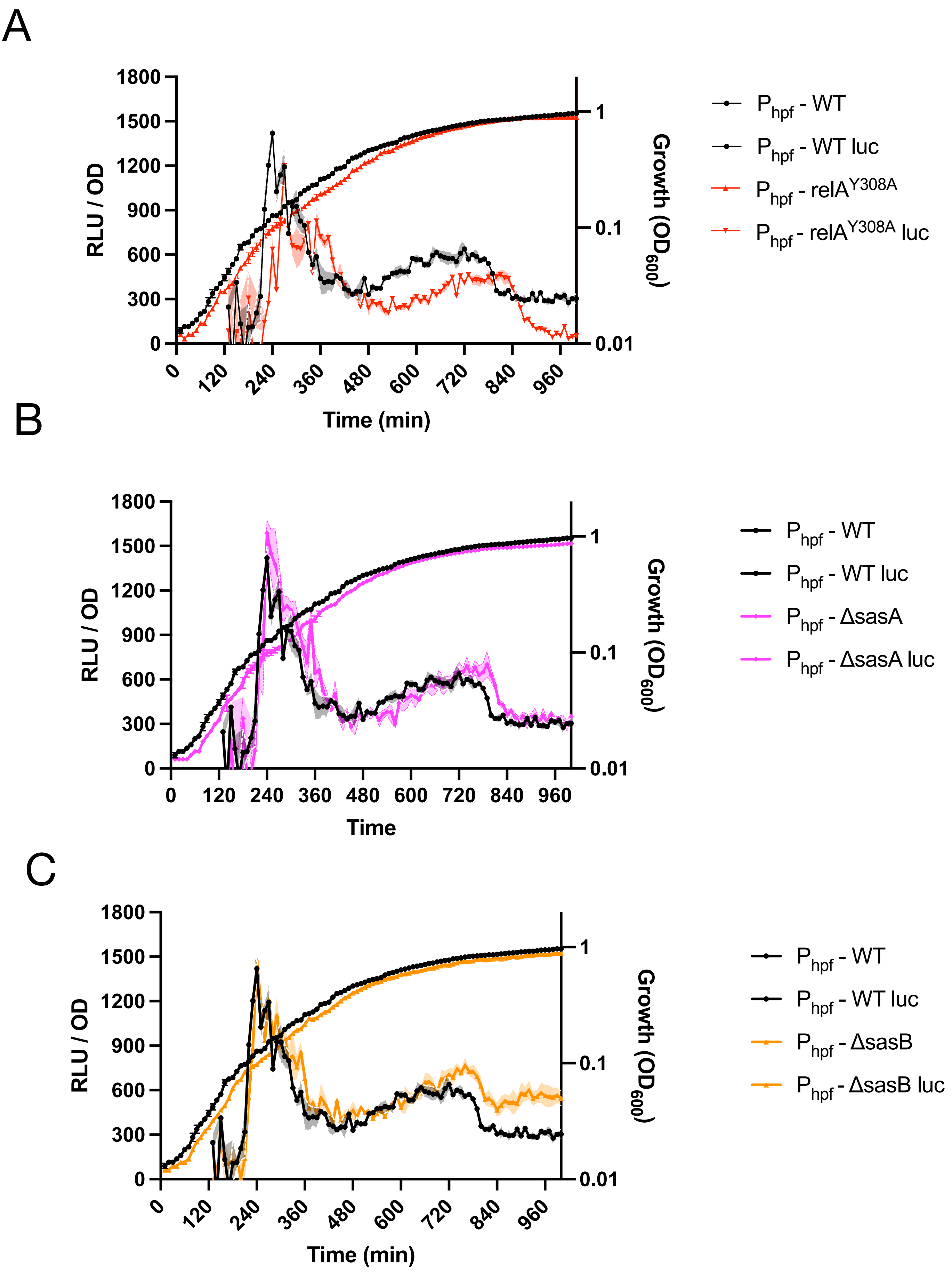

Supplemental Figure S2

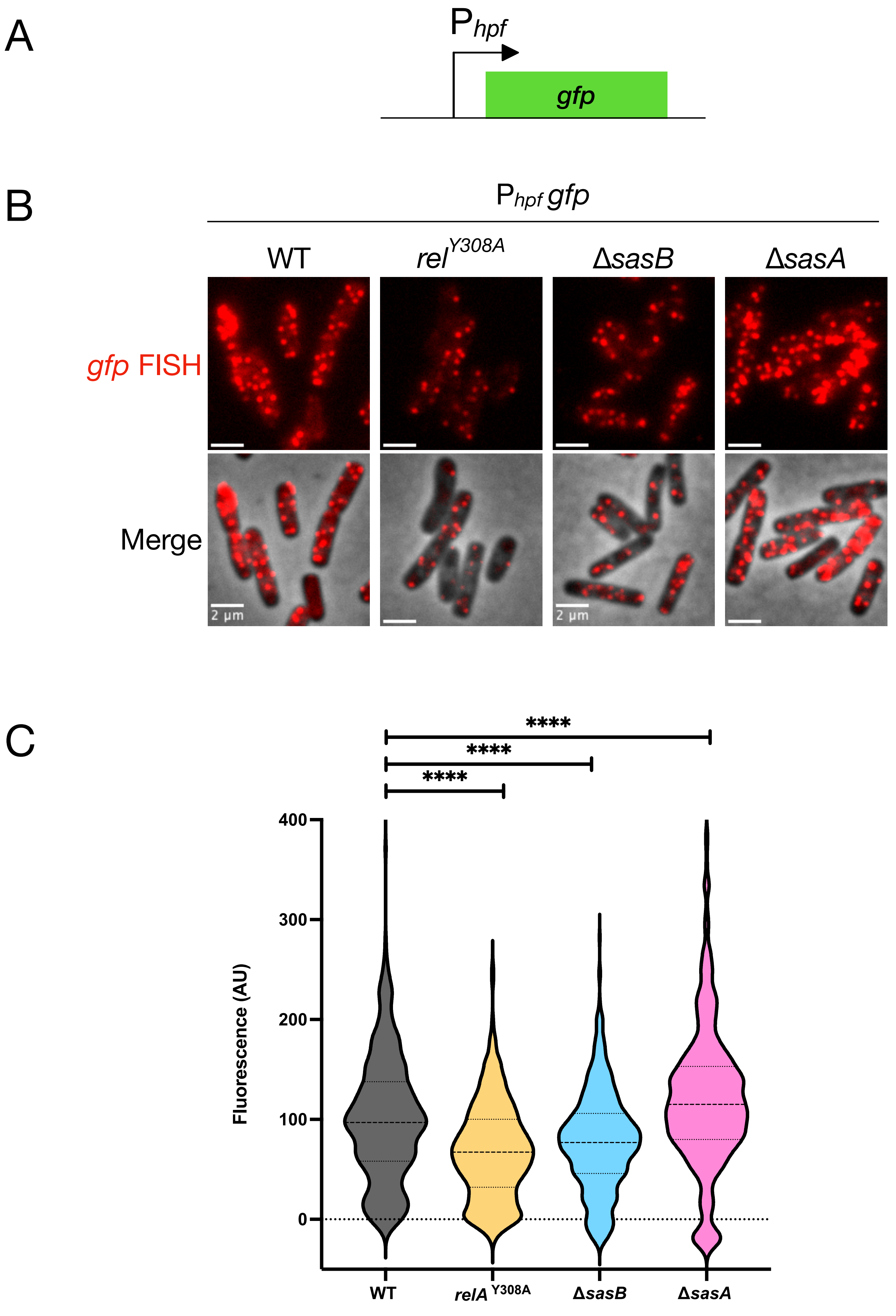

Supplemental Figure S3

A

codY σB-35 σH-35 σB-10 σH-10

AAGTTCACTGAATTTTCACAAAAGATTTATGTTTCAGCAGGAATTGTAAAGGGTAAAAGAGAAATAGATACATATCCTTAATAAAAG P_{hpf}

AAGTTCACTGAATTTTCACAAAAGATTTATaTccCAGCAGGAATTGTAAAGaaTtAAAGAGAAATAGATACATATCCTTAATAAAAG $P_{hpf\text{-}sigB*}$

B

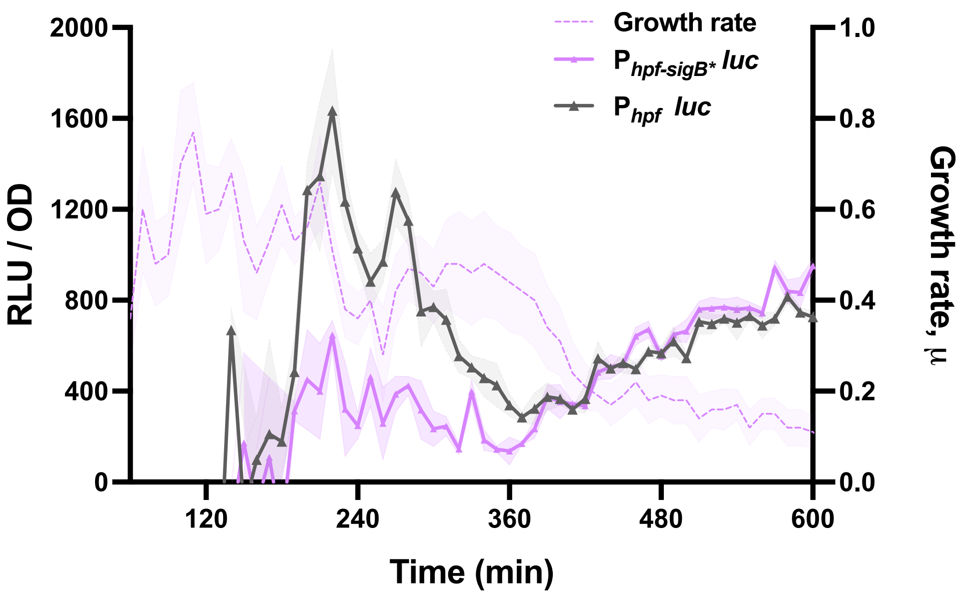

Supplemental Figure S4

A

codY σB-35 σH-35 σB-10 σH-10

AAGTTCACTGAATTTTCACAAAAGATTTATGTTTCAGCAGGAATTGTAAAGGGTAAAAGAGAAATAGATACATATCCTTAATAAAAG P_{hpf}

AAGTTCACTGAATTTTCACAAAAGATTTATaTccCAGCAttAgaaGTAAAGaaTtAAAGAcAgtTgGATACATATCCTTAATAAAAG $P_{hpf\text{-}sigHB*}$

B

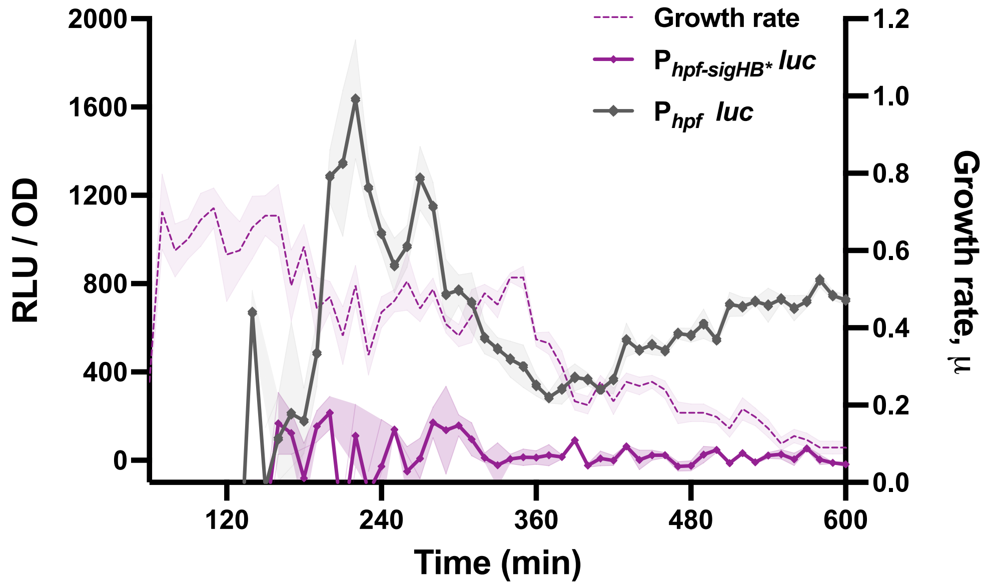

Supplemental Figure S5

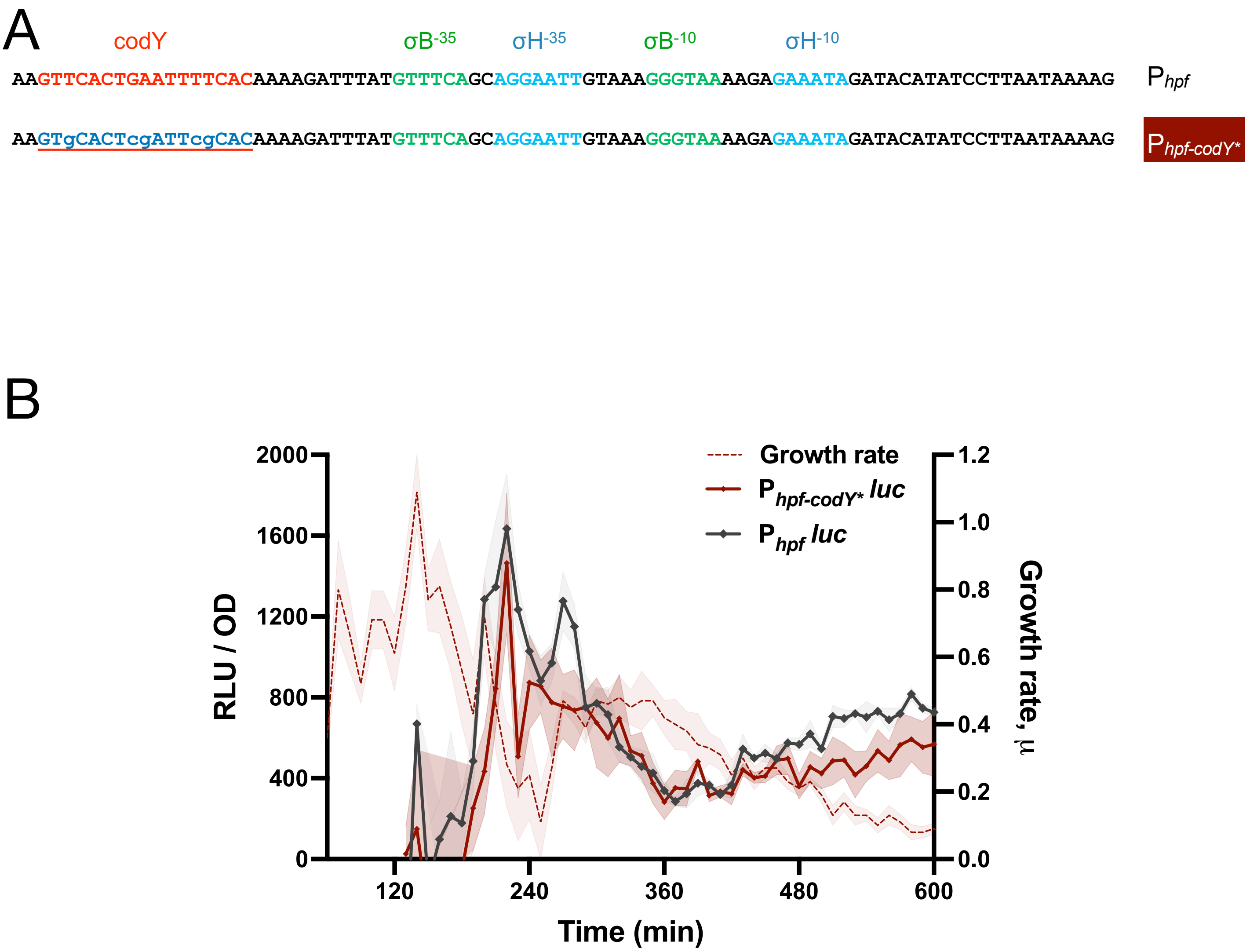

Supplemental Figure S6

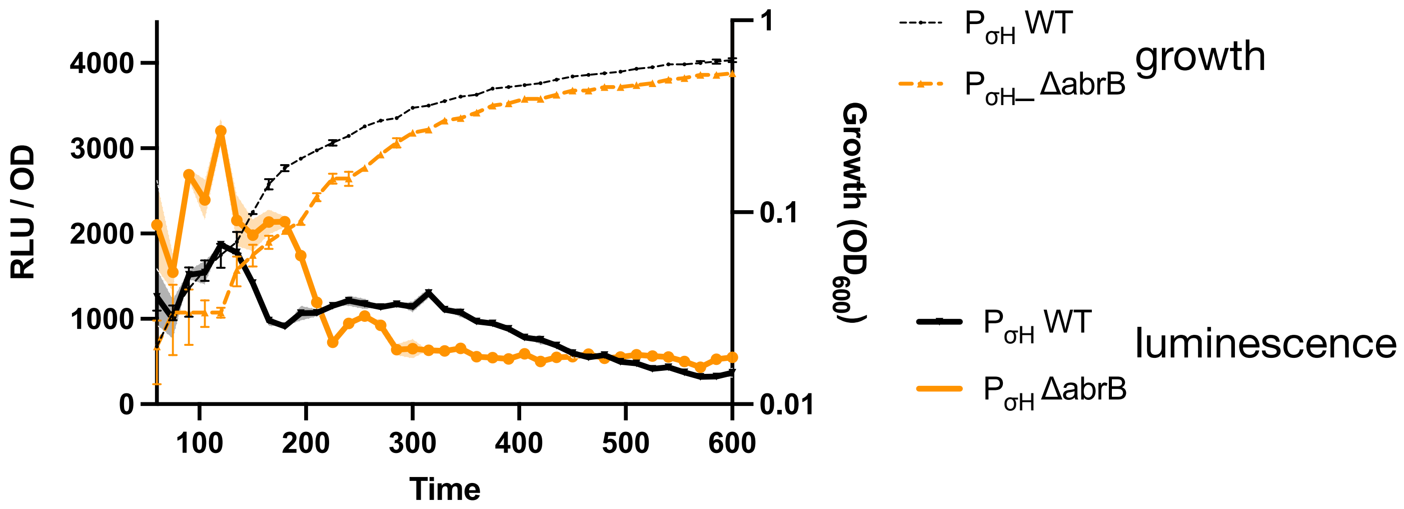

Supplemental Figure S7

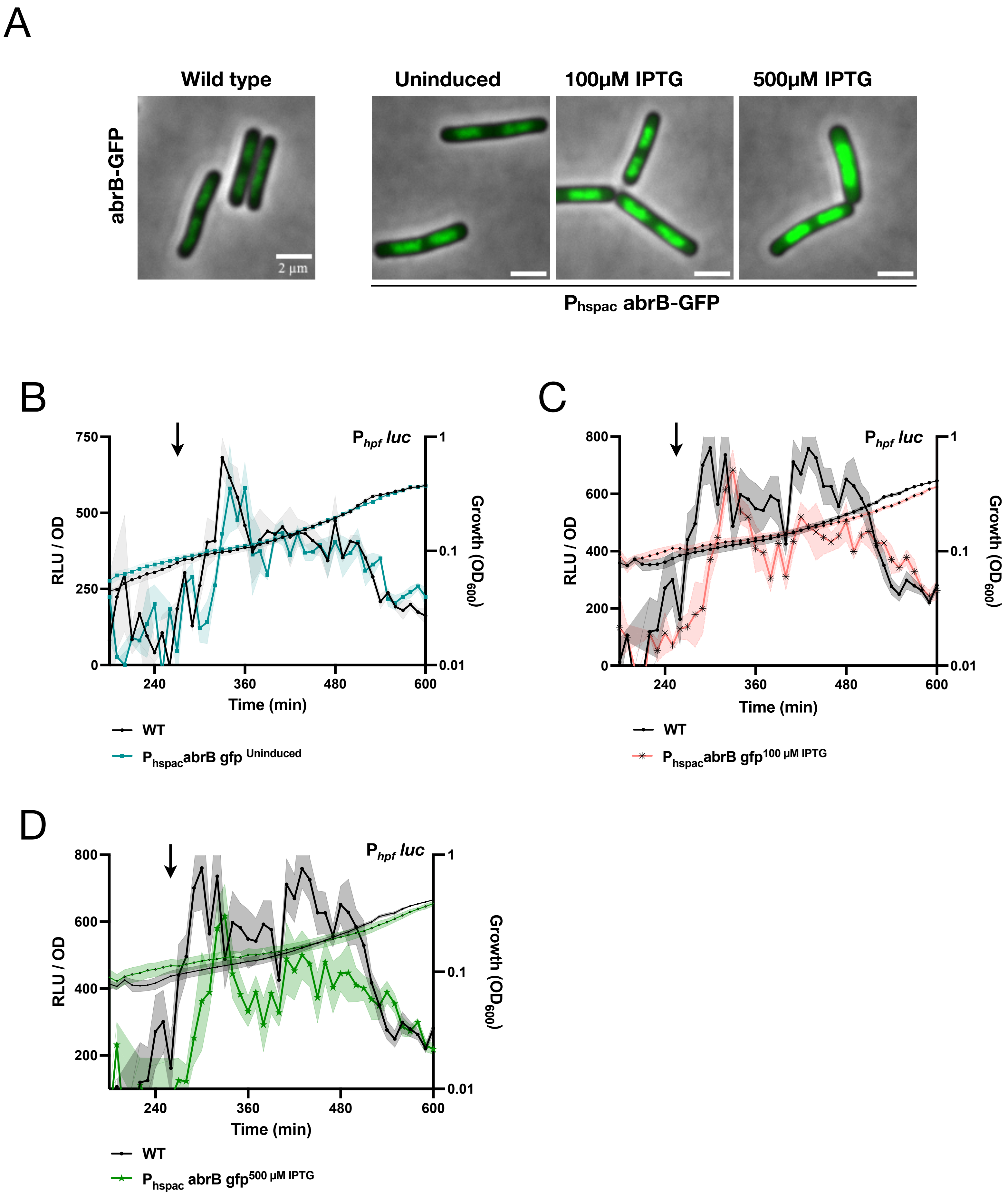

Supplemental Figure S8

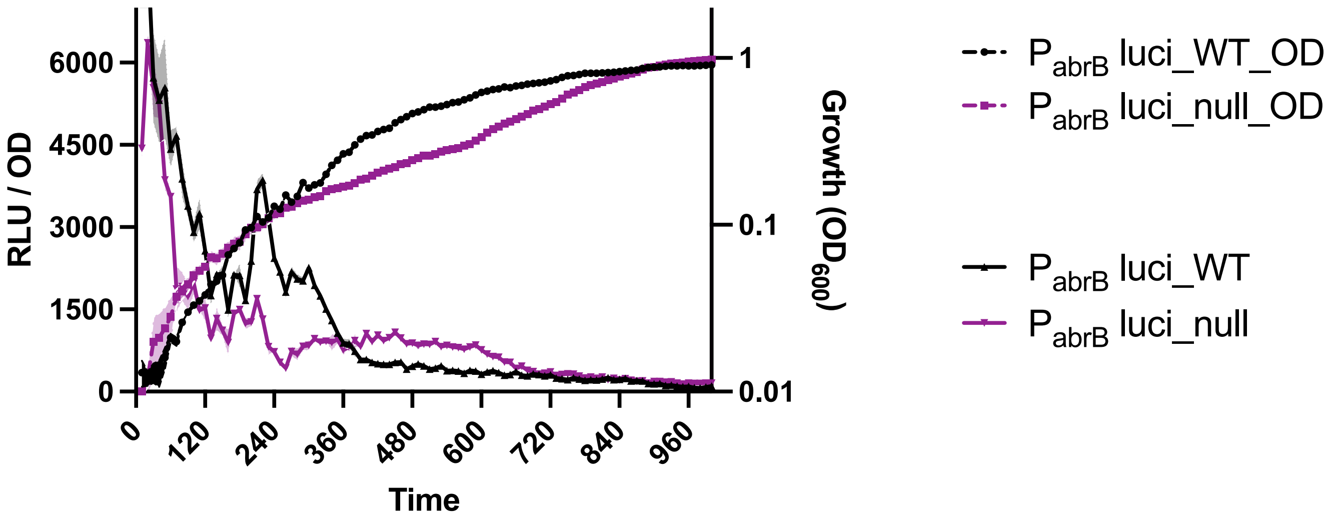

Supplemental Figure S9

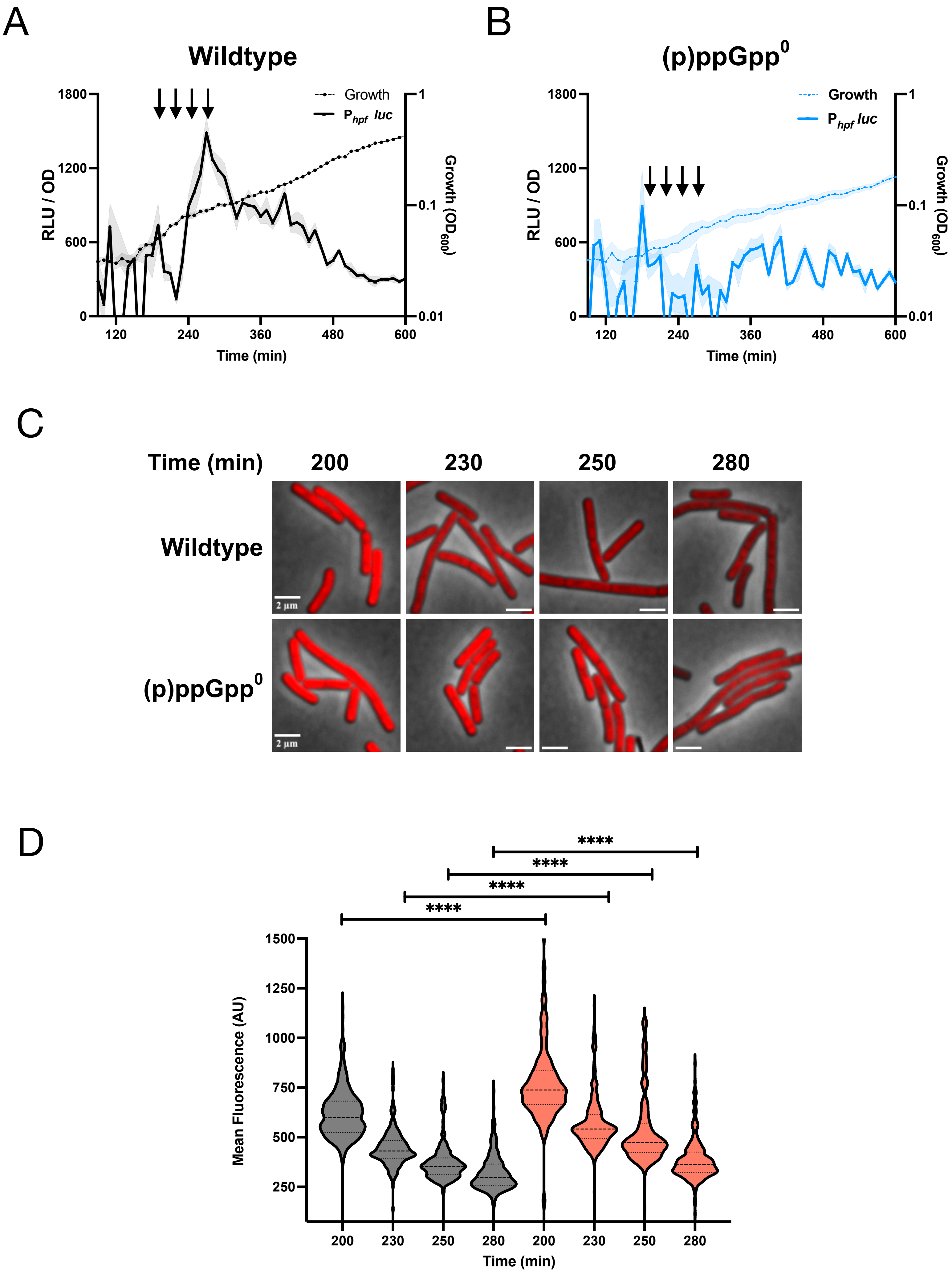

Supplemental Figure S10

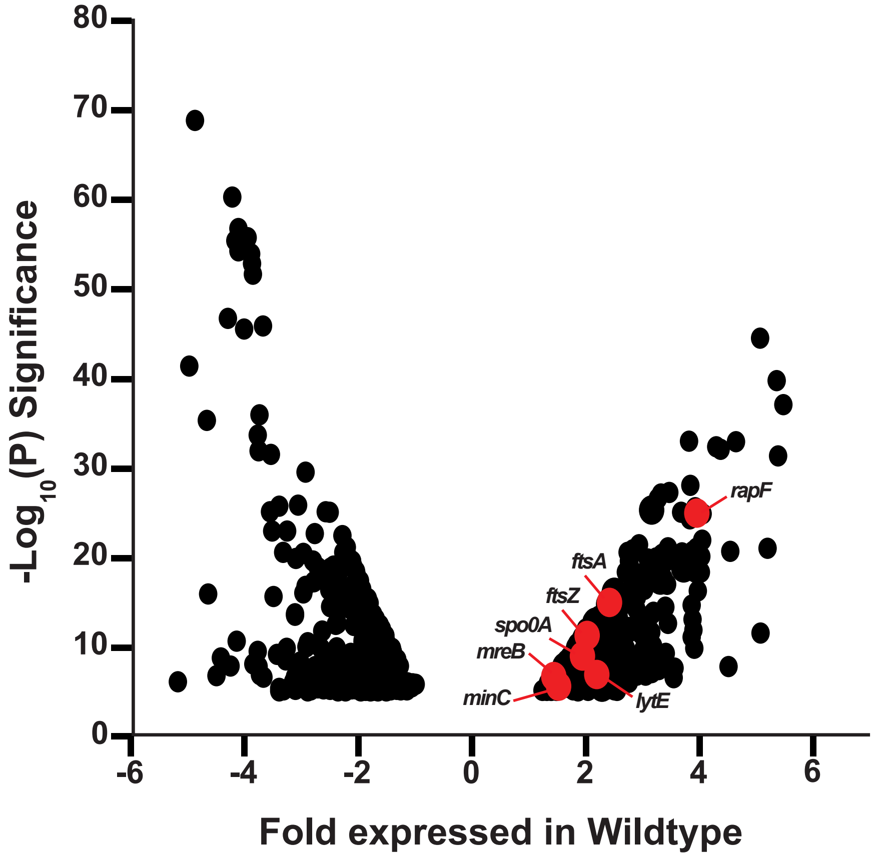

Supplemental Figure S11

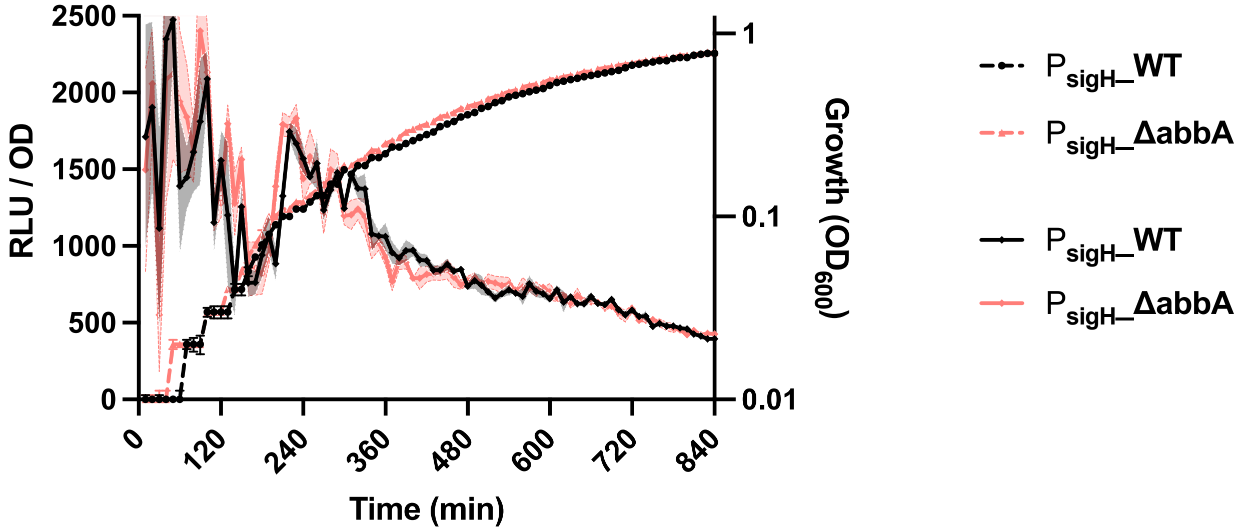

Supplementary Figure Legends

Figure S1. Effects of individual (p)ppGpp synthases on P_{hpf}-luc activity. Growth (OD_{600}) and luminescence (RLU/OD_{600}) measured of: **A,** wildtype (black, JDB4811) and catalytically inactive *relAY308A* (red, JDB4816); **B,** wildtype (black) and *ΔsasA* (violet, JDB4817); and **C,** wildtype (black) and *ΔsasB* (orange, JDB4818) strains. Shown are representatives of at least three biological replicates.

Figure S2. Differential regulatory effects of Rel, SasA, and SasB on P_{hpf} activity assayed by FISH. A, schematic of P_{hpf}-*gfp* fusion. **B**, aliquots of wildtype (JDB4450), *relAY308A*(JDB4825), ∆*sasB* (JDB4827) or ∆*sasA* (JDB4826) strains expressing P*hpf*-*gfp* were collected at t=240 min and probed by FISH*.* The upper panel shows representative fluorescence images of the wild-type and mutant strains, where *gfp* mRNA is hybridized with oligonucleotide probes that specifically bind to it. The red foci indicate the hybridized *gfp* mRNA molecules. Additionally, the lower panel includes merged images with phase contrast. (Scale bar: 2 μm)*.* **C**, population distribution of mean fluorescence (measured in arbitrary units-AU) with *gfp* mRNA-specific probes in the wild type and the mutants. **** denotes a two-tailed P-value, derived from a non-parametric Mann-Whitney test, of $p<0.0001$. The results are representative of three independent experiments.

Figure S3. The regulatory role of SigB in P_{hpf} activity. A, sequence of the *hpf* (top) and the scrambled *hpf*-*sigB** (bottom) promoters. **B,** growth rate, μ (in purple) and luminescence (RLU/OD_{600}) of strains carrying P_{hpf} *luc* (black, JDB4811) and $P_{hpf-sigB*}$ *luc* (purple, JDB4814) reporters. Shown is a representative of at least three biological replicates.

Figure S4. The regulatory role of SigB and SigH in P_{hpf} activity. A, sequence of the *hpf* (top)

and the scrambled *hpf-sigHB** (bottom) promoters. **B,** Growth rate, μ (in purple), and luminescence (RLU/OD_{600}) of strains carrying P_{hpf} *luc* (black, JDB4811) and $P_{hpf\text{-}sigHB*}$ *luc* (purple, JDB4815) reporters. Shown is a representative of at least three biological replicates.

Figure S5. The regulatory effect of CodY on P_{hpf} activity. A, sequence of the *hpf* (top) and the scrambled *hpf-codY** (bottom) promoters. **B,** growth rate, μ (brown) and luminescence (RLU/OD_{600}) of strains carrying P_{hpf} *luc* (black, JDB4811) and P*hpf*-$_{codY*}$ *luc* (brown, JDB4837) reporters. Shown is a representative of at least three biological replicates.

Figure S6. Deletion of the transcriptional regulator AbrB leads to elevated P_{sigH} activity. Growth (OD_{600}) and luminescence (RLU/OD_{600}) of strains carrying P_{sigH} *luc* in wildtype (black, JDB4819) and Δ*abrB* (orange, JDB4821) backgrounds. Shown is a representative of at least three biological replicates.

Figure S7. Overexpressing AbrB reduces HPF induction. **A,** representative fluorescence microscopy image of the wild-type strain (JDB4823) harboring P_{hpf} *luc* expressing endogenous AbrB-GFP fusion (left panel), and the merodiploid strain expressing IPTG-inducible AbrB-GFP (JDB4849), alongside P_{hpf} *luc* and the endogenous *abrB-GFP* (right panel). For AbrB-GFP induction, the inducer was added at t = 0 minutes. The cells were imaged at t_{260} (minutes), marked by arrows in the graphs shown in figures **B-D**. (Scale bar: 2μm). **B-D,** Measuring the luminescence (RLU/OD_{600}) of P_{hpf} *luc* and the growth (OD_{600}) of the wild-type strain (JDB4823), as well as the merodiploid strain expressing IPTG-inducible AbrB-GFP (JDB4849). The black graph line in graphs **B-D** represents the luminescence (RLU/OD_{600}) and growth (OD_{600}) of the wild-type strain (JDB4823). The teal green graph line in **B** shows the luminescence (RLU/OD_{600}) and growth (OD_{600}) of the merodiploid strain (JDB4849) grown without inducer. The orange graph line in **C** shows the luminescence (RLU/OD_{600}) and growth (OD_{600}) of the merodiploid strain (JDB4849)

grown with 100 µM IPTG, while the green graph line in **D** shows the luminescence (RLU/OD$_{600}$) and growth (OD$_{600}$) of the merodiploid strain (JDB4849) grown with 500 µM IPTG. The shading around each graph line represents the SEM, calculated from triplicate measurements.

Figure S8. P$_{abrB}$ activity in a (p)ppGpp^0 strain. Growth (OD$_{600}$) and luminescence (RLU/OD$_{600}$) of strains carrying P$_{abrB}$-luc in wildtype (black, JDB4835) and (p)ppGpp^0 (purple, JDB4836) backgrounds. Shown is a representative of at least three biological replicates.

Figure S9. (p)ppGpp^0 mutant exhibits higher levels of global protein synthesis. A, luminescence (RLU/OD$_{600}$; black) and growth (black) of wildtype strain (JDB4823) harboring P$_{hpf}$ *luc* expressing the AbrB-GFP fusion, and **B,** luminescence (RLU/OD$_{600}$; blue) and growth (blue) of (p)ppGpp^0 strain (JDB4824) harboring P$_{hpf}$ *luc* expressing the AbrB-GFP fusion**.** The shading around the graph line represents the SEM, calculated from triplicate measurements **C,** aliquots from wildtype and (p)ppGpp^0 cultures were collected at time points shown in A, B (black arrows) and labelled with OPP. Shown are representative images of wildtype and (p)ppGpp^0 mutant strains labelled with OPP at the specified time(min). **D,** population distribution of fluorescence upon OPP labelling in wildtype and (p)ppGpp^0 strains at the specified time, shown in 'C'. **** denotes a two-tailed P-value, derived from a non-parametric Mann-Whitney test, of P<0.0001. Results are representative of three independent experiments.

Figure S10. Differential gene expression of wildtype and (p)ppGpp^0 mutant strains. The volcano plot illustrates differential gene expression between the wildtype (JDB4811) and a (p)ppGpp^0 mutant (JDB4812), with each dot representing a single gene. Indicated are *rapF. ftsA, ftsZ, spo0A, mreB, minC, and lytE in* red. The fold differences in gene expression are indicated on the x-axis and the fold difference Log$_{10}$(P) statistical significance is represented on the y-axis.

Figure S11. Effect of AbbA on P_{sigH}-luc. Growth (OD_{600}) and luminescence (RLU/OD_{600}) of strains expressing P_{sigH}-luc in wildtype (black, JDB4819) and Δ*abbA* (pink, JDB4822) backgrounds. Shown is a representative of at least three biological replicates.

Supplementary Tables

Table S1. Strains

Strain	Genotype	Source
JDB1772	*trpC2*	Lab collection
JDB3652	*relA::erm yjbM::tet ywaC::kan codY::spec* P_{spo0A}*-luc*	[20]
JDB4811	*sacA::*P_{hpf}-luc cm^R	This study
JDB4812	*sacA::*P_{hpf}-luc Cm^R, (p)ppGpp^0 *(relA* Y308A; ywaC::kan, yjbM::tet).*	This study
JDB4813	*sacA::*P_{hpf}-*σH**$_{*}$-luc cm^R (scrambled σ^H binding site in P_{hpf})	This study
JDB4814	*sacA:::*P_{hpf}-*σB**-luc cm^R (scrambled σ^B binding site in P_{hpf})	This study
JDB4815	*sacA::*P_{hpf}-*σHB**$_{*}$-luc cm^R (scrambled σB & σH binding site in P_{hpf})	This study
JDB4816	*sacA::*P_{hpf}-luc cm^R*, relA* Y308A*	This study
JDB4817	*sacA::*P_{hpf}-luc cm^R, Δ*ywaC(sasA)::kan*	This study
JDB4818	*sacA::*P_{hpf}-luc cm^R, Δ*yjbM(ΔsasB)::tet*	This study
JDB4819	*sacA::*P_{sigH}-luc cm^R	This study
JDB4820	*sacA::*P_{sigH}-luc cm^R*, relA* Y308A; ywaC::kan, yjbM::tet.*	This study
JDB4821	*sacA::*P_{sigH}-luc cm^R*, abrB::erm*	This study
JDB4822	*sacA::*P_{sigH}-luc cm^R*, abbA::kan*	This study
JDB4534	*pYG1 chr::erm*^{R} P_{wt} *abrB-gfp,* P_{spac} *'abrB*	[29]
JDB4823	*sacA::*P_{hpf}-luc cm^R, P_{abrB}*-abrB-gfp* erm^R	This study
JDB4824	*sacA::*P_{hpf}-luc cm^R, P_{abrB}*-abrB-gfp* erm^R (p)ppGpp^0*(relA* Y308A ywaC::kan yjbM::tet)*	This study
JDB4450	*pyrD::*P_{hpf}*-gfp*^{mut2}*-Cm*^{R}	This study
JDB4825	*pyrD::*P_{hpf}*-gfp*^{mut2}*-Cm*^{R} *relA* Y308A*	This study
JDB4826	*pyrD::*P_{hpf}*-gfp*^{mut2}*-Cm*^{R} Δ*ywaC(sasA)::kan*	This study
JDB4827	*pyrD*::P_{hpf}-gfp^{mut2}-Cm^R Δ*yjbM(ΔsasB)::tet*	This study
JDB4828	*pyrD::*P_{hpf}*-gfp*^{mut2}*-Cm*^{R} (p)ppGpp^0 *(relA* Y308A ywaC::kan yjbM::tet)*	This study
JDB4829	*sacA::*P_{ftsAZ}-luc cm^R	This study
JDB4830	*sacA::*P_{ftsAZ}-luc cm^R (p)ppGpp^0 *(relA* Y308A ywaC::kan yjbM::tet)*	This study
JDB4831	*sacA::*P_{rapF}-luc cm^R	This study
JDB4832	*sacA::*P_{rapF}-luc cm^R (p)ppGpp^0 *(relA* Y308A ywaC::kan yjbM::tet)*	This study
JDB4833	*sacA::*P_{spo0A}-luc cm^R	This study
JDB4834	*sacA::*P_{spo0A}-luc cm^R (p)ppGpp^0 *(relA* Y308A ywaC::kan yjbM::tet)*	This study
JDB4835	*sacA::*P_{abrB}-luc cm^R	This study
JDB4836	*sacA::*P_{abrB}-luc cm^R (p)ppGpp^0 *(relA* Y308A ywaC::kan yjbM::tet)*	This study
JDB4837	*sacA:::*P_{hpf}-*codY**-luc cm^R (scrambled CodY binding site in P_{hpf})	This study
JDB4849	*sacA*::P_{hpf}-luc-cm^R, P_{abrB}-abrB-gfp-erm^R, *amyE*::P_{hyspac} abrB GFP-$spec^R$	This study
JDB4850	*sacA*::P_{sigH}-YFP-Cm^R	This study

JDB4851	*sacA*::P_{hpf}-YFP-Cm^R	This study
JDB4692	*amyE*::P_{veg}-mCherry-$spec^R$	Lab collection

Table S2. Plasmids

Plasmid	JDE	Reference
pSD47 (pSac-cm-P_{veg}-luc)	JDE 3175	[19]
pSN09 P_{hpf} luc	JDE 3305	This study
pSN10 $P_{hpf\text{-}codY*}$ luc	JDE 3306	This study
pSN11 $P_{hpf\text{-}sigB*}$ luc	JDE 3307	This study
pSN12 $P_{hpf\text{-}sigH*}$ luc	JDE 3308	This study
pSN13 $P_{hpf\text{-}sigBH**}$ luc	JDE 3309	This study
pSN21 P_{sigH} luc	JDE 3310	This study
pSN23 P_{abrB} luc	JDE 3311	This study
pSN24 P_{ftsAZ} luc	JDE 3312	This study
pSN28 P_{rapF} luc	JDE 3313	This study
pSN40 P_{hyspac} abrB GFP	JDE 3318	This study
pSN32 P_{sigH} yfp	JDE 3319	This study
pSN55 P_{hpf} yfp	JDE 3321	This study

Table S3. Oligonucleotides

Primer	Name	sequence 5'-3'
NSN 107	pSD47 R	ggatcctgagcgccggtcgctac
NSN 108	pSD47 F	aagcttggcattccggtactgtagg
NSN 109	$P_{hpf/hpf**}$ F	ccggcgctcaggatcccctccgaaccgccaataacag
NSN 110	$P_{hpf/hpf**}$ R	accggaatgccaagctt cttttattaa ggatatgtat c
NSN 122	P_{sigH} F	ccggcgctcaggatccagagaggtagaaacgattgaaaggcg
NSN 123	P_{sigH} R	accggaatgccaagcttcacgtagatagaaatattatacagtattgg
NSN 133	P_{abrB} F	ccggcgctcaggatccaatgctgttatttcggtagtttcc
NSN 134	P_{abrB} R	ccggaatgccaagcttagagatacttatttgtttaaatta
NSN 135	P_{ftsA} F	ccggcgctcaggatcccaataagtttagcttttctggg
NSN 136	P_{ftsA} R	accggaatgccaagcttacatttcgatcatttctattc
NSN 149	P_{rapF} F	ccggcgctcaggatccatcaacgacaaacccttcag
NSN 150	P_{rapF} R	accggaatgccaagctttaattctatatatgtcatattttatcatc
NSN 172	abrB F	gactgaagctttaaggaggaaaaaaaatgtttatgaaatctactggtattgtac
NSN 173	abrB R	agcttgcatgcctatttgtatagttc
NSN 162	*yfp* F	tgactgaagcttataaggaggtcaaaaatggttagc
NSN 163	*yfp* R	cagtctgaattctcttttcggttttaaagaaaaagg
NSN 179	*yfp* R	tgacagaattcctagtatttatacagttcatccatg
